# Scent of death: Evolution from sea to land of an extreme collective attraction to conspecific death

**DOI:** 10.1002/ece3.4912

**Published:** 2019-02-10

**Authors:** Leah Valdes, Mark E. Laidre

**Affiliations:** ^1^ Department of Biological Sciences Dartmouth College Hanover New Hampshire

**Keywords:** collective behavior, death, evolutionary innovation, sea to land, shells, sociality

## Abstract

All living organisms must eventually die, though in some cases their death can bring life‐giving opportunities. Few studies, however, have experimentally tested how animals capitalize on conspecific death and why this specialization would evolve. Here, we conducted experiments on the phylogenetically most closely‐related marine and terrestrial hermit crabs to investigate the evolution of responses to death during the sea‐to‐land transition. In the sea, death of both conspecifics and heterospecifics generates unremodeled shells needed by marine hermit crabs. In contrast, on land, terrestrial hermit crabs are specialized to live in architecturally remodeled shells, and the sole opportunity to acquire these essential resources is conspecific death. We experimentally tested these different species’ responsiveness to the scent of conspecific versus heterospecific death, predicting that conspecific death would have special attractive value for the terrestrial species. We found the terrestrial species was overwhelmingly attracted to conspecific death, rapidly approaching and forming social groupings around conspecific death sites that dwarfed those around heterospecific death sites. This differential responsiveness to conspecific versus heterospecific death was absent in marine species. Our results thus reveal that on land a reliance on resources associated exclusively with conspecifics has favored the evolution of an extreme collective attraction to conspecific death.

## 1 INTRODUCTION

Living things must eventually die. However, from an evolutionary perspective, death creates new opportunities for life (Anderson, Pettitt, & Biro, 2018). Once an organism dies, the resources it previously monopolized become available for exploitation by others (Dawkins, 1976). Resources unlocked by death come in several forms, including biological, physical, and social resources. Biological resources are released as detritivores or scavengers exploit the dead as a food resource, consuming nutrients and energy stored in the chemical bonds of the dead organism's tissue (King, Bailey, Priede, & Browman, 2007). Physical resources released by death are even more varied, including, for example, access to habitat openings and sunlight when a fallen tree creates a canopy gap where other plants can grow (Schaetzl, Burns, Johnson, & Small, 1988), and also cases of resource inheritance, for example, when social insects inherit the nest of a deceased queen (Leadbeater, Carruthers, Green, Rosser, & Field, 2011). Finally, the opportunities produced by death are not exclusively material: The death of socially dominant individuals can also open positions within the dominance hierarchy for subordinates (East & Hofer, 2001). Death, by releasing resources, can thus be a starting point for new life.

How and why organisms tap the potential resources created by death may depend on many factors. Indeed, an organism's death should mean different things to different individuals, depending on several factors: whether individuals belong to the same or different species, what resources are released by death, what resource needs the living individual has, and the costs and benefits of exploiting these resources (Swift & Marzluff, 2015). For mobile organisms, the decision of whether to approach or avoid death sites involves weighing trade‐offs between potential costs and benefits (Sun, Haynes, & Zhou, 2018). Potential costs of approaching death sites include the energy expended in approaching as well as the danger of experiencing predation, injury from fellow conspecifics, or pathogen transmission at the site. Potential benefits include the chance of successful resource and information acquisition (Swift & Marzluff, 2018). The balance between these costs and benefits may differ dramatically for different species specialized for living in different environments. Ultimately, evolution will favor organisms that react to death in ways that maximize reproductive success within their species‐typical environment.

The evolutionary transition from sea to land represents perhaps one of the most dramatic changes in environment (Vermeij, 2017), potentially exerting a strong influence on responses to death (Gonçalves & Biro, 2018). For species that successfully transitioned from sea to land, it was essential to adapt to radically different constraints and opportunities. New selective pressures on land ultimately led to a variety of evolutionary innovations, many of which had never appeared before in the sea (Vermeij, 2017). With respect to death, organisms from sea‐dwelling and land‐dwelling species both inevitably die, but the frequency of death, the utility of the resources released through death, and the potential risk of seeking out such death likely vary between sea and land. For shell‐bearing organisms, in particular, the predation pressures that are a major cause of death are much stronger in the sea than on land (Vermeij, 1993). Moreover, for organisms that use an olfactory system to sense death from a distance, the opportunities for detecting death also differ between sea and land (Harzsch & Krieger, 2018), with relevant odorants in marine environments being predominantly hydrophilic, while relevant odorants on land are generally hydrophobic and volatile (Krang, Knaden, Steck, & Hannson, 2012). Finally, and perhaps most critically from an evolutionary standpoint, the potential information (Dall, Giraldeau, Olsson, McNamara, & Stephens, 2005) contained in death‐related cues, and also the fitness consequences of approaching deaths sites, may differ starkly on land versus in the sea, shifting the optimal responses for terrestrial compared to marine species. Contrasting species from these two environments may therefore yield insights into how and why environmental context has shaped evolutionary specializations and adaptations in responding to death.

Hermit crabs provide a powerful system for investigating evolved responses to death in terrestrial versus marine environments. These animals embody the life‐giving properties of death: Both marine and terrestrial species are obligate inhabitants of shells, which are vital for survival, growth, and reproductive success; and these shell resources only become available via death (Laidre, 2011), either of the original shell‐building gastropod (Rittschof, 1980; Valdes & Laidre, 2018) or of a fellow hermit crab (Laidre, 2012a; Small & Thacker, 1994). Ancestrally, all hermit crabs were marine‐dwelling, but one family in particular (Coenobitidae) has become evolutionarily specialized to live on land (Bracken‐Grissom et al., 2013). Critically, these terrestrial hermit crabs face different selective pressures than marine hermit crabs in acquiring shells following death. Marine hermit crabs are specialized to live in unremodeled shells, which can derive either from the death of a gastropod or of any marine hermit crab (Laidre & Trinh, 2014), making both heterospecific and conspecific death equally significant for these marine species. In contrast, such unremodeled shells are effectively unusable by terrestrial hermit crabs (Laidre, 2012a). Instead, terrestrial hermit crabs are specialized to live in shells that have been architecturally remodeled by conspecifics (Laidre, 2014). Remodeled shells, which are reused and passed down across generations, can only be acquired following the death (or eviction) of a conspecific (Laidre, 2018a), making conspecific death, but not heterospecific death, of unique significance for these terrestrial species. Given the differing modes of shell acquisition for terrestrial and marine hermit crabs, a key prediction is that the relative attractive value of conspecific versus heterospecific death should have diverged evolutionarily between these species.

Here, we investigated the evolved specializations by which organisms on land versus those in the sea exploit death to acquire life‐giving resources. In field experiments, we contrasted responses between the phylogenetically most closely‐related marine and terrestrial hermit crab species, testing the salience of the scent of conspecific versus heterospecific death. First, for both marine and terrestrial hermit crabs, we tested the relative attractiveness of the death of conspecifics versus the death of heterospecifics (gastropods). Then, for terrestrial hermit crabs, we examined an even finer‐grained comparison, testing the relative attractiveness of the death of conspecifics versus closely‐related heterospecifics (marine hermit crabs). We predicted that both terrestrial and marine hermit crabs would exploit the scent of death to locate sites of shell availability. However, given the exclusive reliance of terrestrial—but not marine hermit crabs—on shells derived from conspecific death, we predicted that only terrestrial hermit crabs would exhibit a differential and overwhelming collective attraction to the scent of conspecific death.

## 2 MATERIALS AND METHODS

### 2.1 Study site and species

**Figure 1:**
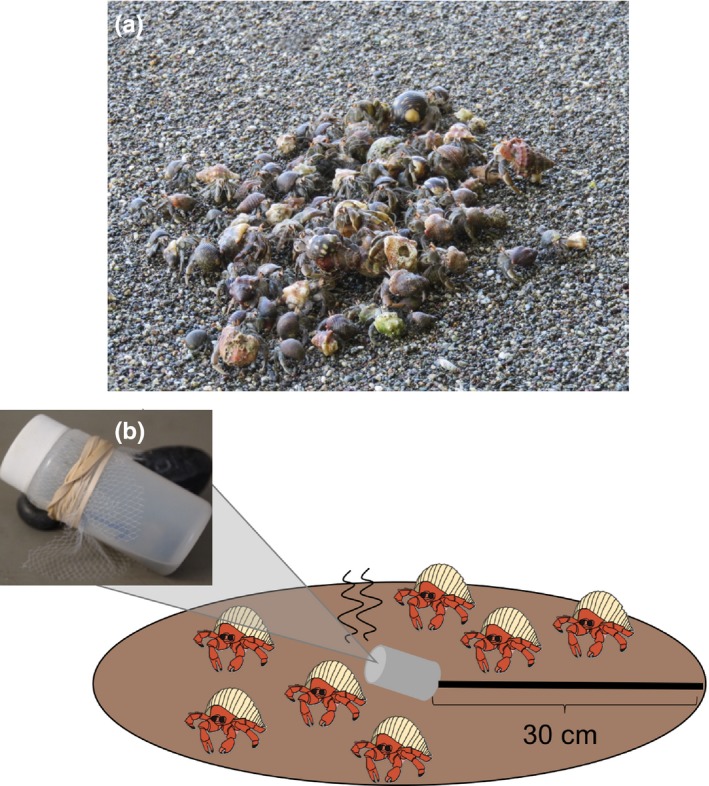
Study species and experimental design. (a) Natural grouping of highly social terrestrial hermit crabs (*Coenobita compressus*), one of the study species. This land‐based species is special, in that it lives in architecturally remodeled shells, which can only be acquired after conspecifics are evicted or die. The scent of conspecific death may therefore generate extreme collective attraction. Photo by Mark Laidre. (b) Experimental design, with a capped bottle containing different chemical cues (including the scent of death) being placed on the substrate and then opened. The same setup was used both on land (with terrestrial hermit crabs, *Coenobita compressus*) and in the sea (with marine hermit crabs, *Calcinus obscurus* and *Clibanarius albidigitus*). The number of hermit crabs within a circular area (30 cm radius of the bottle) was counted both before the bottle was introduced (at *t* = 0 min) and after individuals had an opportunity to be attracted (at *t* = 5 min)

This study was conducted from January to March 2018 at the Piro Biological Station in the Osa Peninsula of Costa Rica, a location that has served as a long‐term field site for hermit crab studies since 2008 (Laidre, 2010). This site contains some of the phylogenetically most closely‐related terrestrial (*Coenobita compressus*) and marine (*Calcinus obscurus* and *Clibanarius albidigitus*) hermit crabs (Bracken‐Grissom et al., 2013; Laidre, 2019). The terrestrial species (Figure 1a) is the only terrestrial hermit crab along the entire Pacific coastline of North and South America. At the study site, the terrestrial species also lives in close proximity to the two marine species, the three being nearly sympatric, with the two marine species co‐occurring within the same tide pools and with the terrestrial species roaming the beach on land immediately adjacent to and around these tide pools. Together, these species thus provided an opportunity to contrast the responses to death of close evolutionary relatives that occupy adjacent environments, in the sea versus on land. The key difference between these species is their shell specialization (Laidre, 2014). All these hermit crab species commonly occupy *Nerita scabricosta* gastropod shells at the study site (Laidre, 2012b), but whereas both marine species are specialized to live in unremodeled versions of these shells (which can be acquired after all types of death: heterospecific gastropod death, heterospecific marine hermit crab death, and conspecific death), the terrestrial species is instead specialized to live in remodeled versions of these shells (which can only be acquired after conspecific death; Laidre, 2012a).

### 2.2 General experimental design

The protocol and experimental procedures employed in this study were reviewed and approved by the Costa Rican Ministry of Environment and Energy as well as our home institution. Experiments were conducted during daylight hours (between 0600 and 1800 hr) within 3 hr of low tide. Both marine and terrestrial experiments were conducted on the same day, in randomized order, generally within 30 m distance of one another and near to Bosque del Cabo (N 08 22.742, W 083 18.263). This area generally had continuous background winds, such that scents should readily have dispersed in both environments during our experiments. For marine experiments, stimuli were provided in tide pools of the rocky intertidal, with given tide pools tested at most once in a 24‐hr period, and only being tested if they contained at least three visible marine hermit crabs. For terrestrial experiments, experimental stimuli were provided on land on the beach sand, with each terrestrial site placed at least 5 m away from any previously tested terrestrial site, and likewise only being tested if there were at least three visible terrestrial hermit crabs in the vicinity. At each chosen site, marine and terrestrial, the number of hermit crabs within a circular area (30 cm radius) was visually counted immediately before the start of the experiment. The experiment proceeded only if this 30 cm radius contained at least one individual. To begin an experiment, a bottle containing one of five different chemical cue conditions (see below) was randomly chosen and placed at the center of the circular area, thereby releasing the chemical cues into the water or air (Figure 1b). The experimenter then left the site, so as to not influence the hermit crabs, and returned after five minutes. At this time, the number of hermit crabs within the same area was recounted to determine the extent to which individuals were attracted to the scent. A total of *N* = 200 experiments were performed (*N* = 100 in the sea for marine hermit crabs and *N* = 100 on land for terrestrial hermit crabs). For both the sea and the land experiments, *N* = 20 replicates were conducted across each of the five conditions (listed below); the sole exception was two conditions in the land experiments (in which there were *N* = 21 and *N* = 19 replicates, respectively).

### 2.3 Experiment 1: Attraction to the scent of death on land versus in the sea

To measure the attraction of marine versus terrestrial species to the scent of death, we provided chemical cues that simulated death sites (see Valdes & Laidre, 2018). All chemical cue conditions were prepared immediately before the start of the experiment. Each condition was isolated inside a 20‐ml opaque plastic bottle that contained 1.5 ml of freshwater. The bottle's screw‐top lid was only opened and removed at the start of the experiment. Fine plastic mesh and a 6 oz. fishing weight were secured to the opening of each bottle with a rubber band to contain the contents and ensure that the bottle remained weighted to the substrate (Figure 1b).

We tested a total of five chemical cue conditions, including two control conditions (involving living organisms or involving enzymes in the absence of any animal flesh) and three experimental death conditions (involving the flesh of freshly killed gastropods or hermit crabs):

1. Trypsin (control): 1.2 mg of trypsin, nothing else added. This is a digestive enzyme used by some predators of shell‐bearing organisms, but which has no ecological significance if not paired with flesh (Valdes & Laidre, 2018).
2. Live gastropod (control): a live (*Nerita scabricosta*) gastropod (1–3 g).
3. Pure gastropod flesh: 0.7–1.3 g of flesh extracted from a freshly smashed (*Nerita scabricosta*) gastropod. This condition simulated the pure chemical cues associated with the death of a gastropod, which indicates unremodeled shell availability.
4. Trypsin‐treated gastropod flesh: This condition was identical to the pure gastropod flesh condition, except that the flesh was treated with 1.2 mg of trypsin. This condition simulated the digested flesh cues associated with the death of a gastropod. Like pure gastropod flesh, this condition also indicates unremodeled shell availability. However, since trypsin is used by predators to digest away flesh, without damaging the shell, this condition simultaneously indicated that the shell was less likely to have been damaged during the gastropod's death (Valdes & Laidre, 2018).
5. Conspecific flesh: 0.7–1.3 g of flesh extracted from a freshly smashed conspecific hermit crab (for experiments in the sea, the smashed individual was a marine hermit crab, *Calcinus obscurus*, the species that was disproportionately most abundant of the two marine species; for experiments on land, the smashed individual was a terrestrial hermit crab, *Coenobita compressus*). This condition simulated the chemical cues associated with conspecific death, which for terrestrial hermit crabs indicates remodeled shell availability and which for marine hermit crabs (irrespective of species) indicates unremodeled shell availability.

No significant difference existed in either the marine or terrestrial experiments in the weight of flesh across the pure gastropod, trypsin‐treated gastropod, and conspecific conditions (marine experiments: one‐way ANOVA: *F*_2,57_ = 0.31, *p* = 0.74; terrestrial experiments: one‐way ANOVA: *F*_2,58_ = 0.18, *p* = 0.84). Note that neither the trypsin control condition nor the live gastropod control condition were included in this comparison, given that they were in their own weight range.

### 2.4 Experiment 2: Attraction on land to conspecific versus closely‐related heterospecific death

This experiment tested terrestrial hermit crabs’ attraction to the scent of conspecific death (i.e., another terrestrial hermit crab, *Coenobita compressus*) versus the scent of closely‐related heterospecific death (i.e., a marine hermit crab, *Calcinus obscurus*). For terrestrial hermit crabs, the death of a conspecific indicates the availability of a remodeled shell, upon which they depend, while the death of a heterospecific (even a closely‐related heterospecific, like a marine hermit crab) indicates the availability of an unremodeled shell, which is effectively unusable. *N* = 40 experiments were conducted (*N* = 20 replicates for both conditions). Sample preparation and procedures for this experiment were identical to those in Experiment 1, except they were carried out exclusively on land and with only the following two chemical cue conditions:

1. Conspecific flesh: 0.7–1.3 g of flesh extracted from a freshly smashed terrestrial hermit crab (*Coenobita compressus*). This condition simulated the chemical cues associated with the death of the inhabitant of a remodeled shell.
2. Closely‐related heterospecific flesh: 0.7–1.3 g of flesh extracted from a freshly smashed marine hermit crab (*Calcinus obscurus*). This condition simulated the chemical cues associated with the death of the inhabitant of an unremodeled shell.

No significant difference existed in the weight of flesh between the conspecific and heterospecific conditions for this experiment (Mean ± *SE* of conspecific flesh: 0.95 ± 0.03 g; heterospecific flesh: 1.03 ± 0.03 g; two‐sample *t* test: *t* = 1.89, *df *= 37.66, *p* = 0.07).

### 2.5 Predictions and statistical analyses

We predicted that both marine and terrestrial hermit crabs would use chemical cues associated with shell‐occupant death to locate sites of potential shell availability, thus being highly attracted to the scent of death relative to the control conditions. However, based on marine and terrestrial hermit crabs specializing on different types of shells (marine on unremodeled and terrestrial on remodeled), and based on the fact that each shell type is only associated with specific death events, we predicted the following:

1. Marine hermit crabs should be equally attracted to chemical cues associated with the death of gastropods and the death of fellow marine hermit crabs (both of which indicate unremodeled shell availability).
2. Terrestrial hermit crabs, in contrast, should be weakly attracted to chemical cues associated with the death of gastropods and with the death of closely‐related heterospecific marine hermit crabs. Instead, terrestrial hermit crabs should be most attracted to the death of conspecifics (which indicates remodeled shell availability).

For Experiment 1, we analyzed marine experiments and terrestrial experiments separately. We first performed one‐way ANOVAs across all conditions in the experiment, both for the “before” (i.e., *t* = 0 min) and “after” (i.e., *t* = 5 min) counts. We next used paired *t* tests to compare the before‐to‐after change in the number of hermit crabs for each of the five chemical cue conditions in the experiment. We then used orthogonal contrast tests to compare the number of hermit crabs that were attracted at *t* = 5 min, specifically contrasting (a) the pure gastropod flesh condition versus the trypsin‐treated gastropod flesh condition, and (b) the conspecific hermit crab flesh condition versus the pure gastropod flesh condition.

For Experiment 2, we used paired *t* tests to compare the before‐to‐after change in the number of hermit crabs for both the conspecific condition and the closely‐related heterospecific condition. We then used a *t* test to compare the number of hermit crabs attracted at *t* = 5 min to the conspecific condition versus the closely‐related heterospecific condition.

All statistics were run in JMP® Pro (version 12.1.0). When undertaking multiple tests on the same data, the Bonferroni method was used to control the overall alpha level at 0.05.

## 3 RESULTS

### 3.1 Experiment 1: Attraction to the scent of death on land versus in the sea

#### 3.1.1 On land

**Figure 2:**
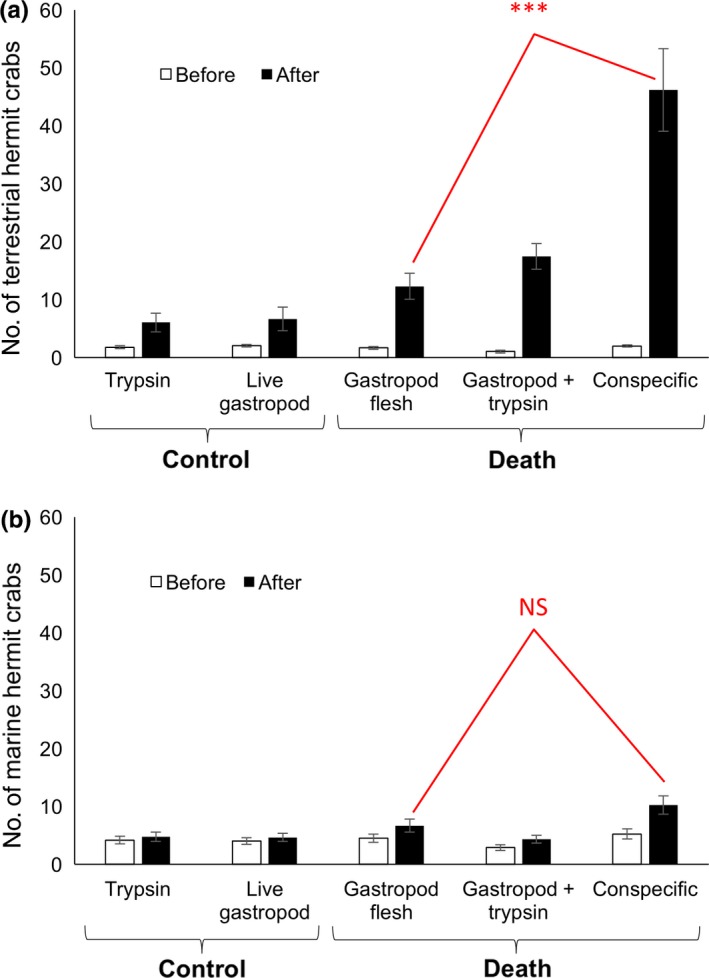
Attraction to the scent of death on land versus in the sea. Number (mean ± *SEM*) of (a) terrestrial hermit crabs (in the experiments on land) and (b) marine hermit crabs (in the experiments in the sea) before and after chemical cues were released. *Y*‐axis is scaled the same for terrestrial and marine. ***p < 0.0001 and NS = nonsignificant

The scent of death was highly attractive on land (Figure 2a; see also Supporting Information Video S1). While there was no significant difference across conditions in the number of terrestrial hermit crabs at *t* = 0 min, there was a significant difference across conditions at *t* = 5 min (see Table 1 for all statistical results). The number of terrestrial hermit crabs did not change significantly from before to after for either of the controls, but did increase significantly for all three experimental death conditions. Contrast tests of the number of terrestrial hermit crabs that accumulated after 5 min showed no difference in attraction between the two gastropod death conditions, but revealed a significantly greater attraction to conspecific death versus gastropod death.

**Table 1:** Attraction to the scent of death

Tests	Terrestrial		Marine	
	Test statistic	*p*‐value	Test statistic	*p*‐value
At *t* = 0 min (One‐way ANOVA)	*F*_4,95_ = 2.89	*p* = 0.0263 (NS)	*F*_4,95_ = 1.66	*p* = 0.16
At *t* = 5 min (One‐way ANOVA)	*F*_4,95_ = 20.10	*p* < 0.0001	*F*_4,95_ = 5.69	*p* = 0.0004
Trypsin control (Paired *t* test)	*t*(18) = 2.77	*p* = 0.0125 (NS)	*t*(19) = 1.49	*p* = 0.15
Live gastropod control (Paired *t* test)	*t*(19) = 2.41	*p* = 0.0264 (NS)	*t*(19) = 1.55	*p* = 0.14
Pure gastropod flesh (Paired *t* test)	*t*(20) = 4.96	*p* < 0.0001	*t*(19) = 3.37	*p* = 0.0032
Trypsin‐treated gastropod flesh (Paired *t* test)	*t*(19) = 7.51	*p* < 0.0001	*t*(19) = 3.96	*p* = 0.0008
Conspecific hermit crab flesh (Paired *t* test)	*t*(19) = 6.16	*p* < 0.0001	*t*(19) = 4.90	*p* < 0.0001
Trypsin‐treated gastropod flesh versus pure gastropod flesh (Contrast test at *t* = 5 min)	*F*_1,95_ = 1.01	*p* = 0.32	*F*_1,95_ = 2.59	*p* = 0.11
Conspecific hermit crab flesh versus pure gastropod flesh (Contrast test at *t* = 5 min)	*F*_1,95_ = 43.27	*p* < 0.0001	*F*_1,95_ = 5.90	*p* = 0.0170 (NS)

Note

Results of statistical analyses from Experiment 1 on terrestrial and marine hermit crabs.

NS = nonsignificant with Bonferroni correction; White cells = significant; gray cells = nonsignificant.

#### 3.1.2 In the sea

In the sea, marine hermit crabs showed the same patterns across all tests as the terrestrial hermit crabs had on land (see above), except for one difference: In marine hermit crabs, there was no significant difference in attraction between conspecific death versus gastropod death (Figure 2b; see Table 1 for all statistical results).

### 3.2 Experiment 2: Attraction on land to conspecific versus closely‐related heterospecific death

**Figure 3:**
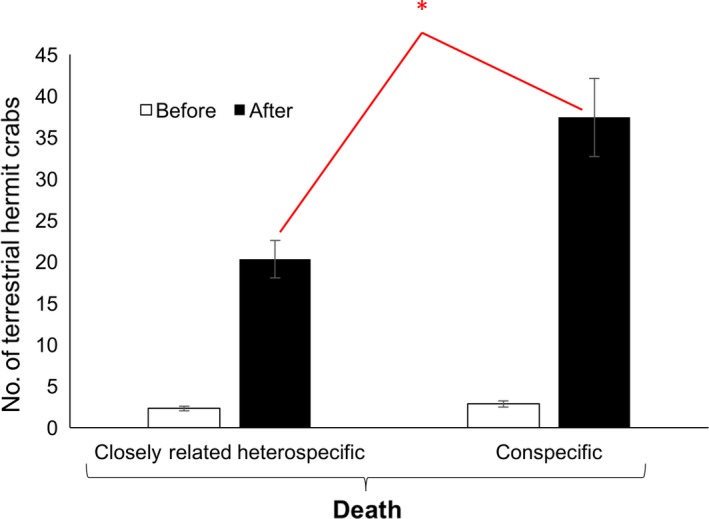
Attraction on land to conspecific versus closely‐related heterospecific death. Number (mean ± *SEM*) of terrestrial hermit crabs before and after chemical cues were released indicating either the death of a closely‐related heterospecific marine hermit crab or the death of a conspecific terrestrial hermit crab. *p < 0.005

The number of terrestrial hermit crabs increased significantly from before to after for both the death of conspecifics and for the death of closely‐related heterospecific marine hermit crabs (Figure 3; paired *t* test for conspecific death: *t* = 7.36, *df *= 19, *p* < 0.0001; for closely‐related heterospecific death: *t* = 7.90, *df *= 19, *p* < 0.0001). Critically though, the accumulation of terrestrial hermit crabs at *t* = 5 min was significantly greater in response to the death of conspecifics versus the death of closely‐related heterospecifics (two‐sample *t* test: *t* = 3.27, *df *= 27.11, *p* = 0.0029).

## 4 DISCUSSION

Death fuels life by unlocking resources (Dawkins, 1976). Some resources, like shells, can long outlast the lifetime of a succession of owners (Vermeij, 1993). Cues associated with death can therefore be exploited by mobile organisms to orient directly to sites where such resources become available. Our results reveal that olfactory cues associated with the death of shell‐bearing organisms can provide information that is highly attractive, and that, furthermore, this attractiveness can vary, both with the shell type of the organism that dies and with the species‐typical environment of the responding organism. In particular, we found stark differences in responses to death by marine versus terrestrial species, which were close phylogenetic relatives. The marine species were equally attracted to conspecific and gastropod death, consistent with both forms of death producing the unremodeled shells that marine species prefer. On the other hand, the terrestrial species was more attracted to conspecific over gastropod death, a difference in response that is consistent with their targeting death sites that yield the architecturally remodeled shells they are specialized to live in. Interestingly, the terrestrial species was not only more attracted to the death of conspecifics compared to the death of distantly related heterospecifics (i.e., gastropods), but was also more attracted to the death of conspecifics compared to the death of closely‐related heterospecifics (i.e., marine hermit crabs). Broadly, these results suggest an evolutionary fine‐tuning during the transition from sea to land, in which selection on land favored an extreme collective attraction to the scent of conspecific death.

Across the animal kingdom, different species manifest a diversity of responses to conspecific death (Anderson et al., 2018). While in many species conspecific death is repellant, eliciting avoidance, other responses include: carrying and disposing of corpses (social insects: Sun et al., 2018); brief inspections followed by alarm calling and mobbing (corvids: Iglesias, McElreath, & Patricelli, 2012; Swift & Marzluff, 2015, 2018); prolonged care and handling of dead infants (nonhuman primates: Biro et al., 2010); and ritualized funerals and mourning (humans: Shimane, 2018). Along the continuum ranging from avoidance to indifference to attraction, what selection pressures would favor an extreme attraction to dead conspecifics? One major factor is likely the value of the resource freed up after death. Species utilizing resources that are rare, highly valuable, and that only become available after a conspecific's death would be expected to exhibit some of the strongest levels of attraction to dead conspecifics. Such criteria are likely met not just in terrestrial hermit crabs, but also in other species in which resource‐limited dwellings, like burrows and other shelters, are costly to construct or are highly saturated (Hansell, 2005; Laidre, 2018a). Under these circumstances, the most cost‐effective strategy may be to take over such resources immediately following a conspecific's death. Broader comparative research could test this “resource‐acquisition through conspecific‐death‐attraction” hypothesis by taking the approach followed here and contrasting phylogenetically closely‐related species, only some of which utilize valuable resources that are liberated after conspecific death.

To respond adaptively to conspecific death, animals must be able to promptly detect death in the first place. While many possible sensory modalities could theoretically function for this purpose (Stevens, 2013), chemical cues are arguably the most effective: Unlike acoustic cues, which are nonexistent in the dead, and also unlike visual cues, which do not transmit at night, chemical cues transmit at all times of day, travel vast distances after death, and can also be differentiated almost immediately, based on highly specific bodily fluids released by the organism that died (Gonçalves & Biro, 2018). In our study, differences in sensory constraints or chemical flow regimes between the two environments (sea and land) might seemingly have contributed to the differential response patterns we observed in marine versus terrestrial species. However, such an explanation is undermined by the extreme sensory specializations, both of the marine and terrestrial species. Indeed, analyses of antennule morphologies in marine and terrestrial crabs (Waldrop, Miller, & Khatri, 2016) have revealed that each is highly adapted to capturing odors within their native habitats, sea and land, respectively. Given how specialized marine and terrestrial species are in morphological hardware for sensing localized chemical plumes within their respective environments, it suggests millions of years of natural selection has ensured optimal sensing and adaptive responses within each of these quite different environments. Interestingly, the chemical sense of Coenobitidae, the terrestrial hermit crab family, is not only highly developed, but also involves a different mechanism of antennule flicking than aquatic crustaceans use (Waldrop & Koehl, 2016), with an unusually large portion of terrestrial hermit crabs’ brains being devoted to this specialized olfactory sensing (Harzsch & Hansson, 2008; Harzsch & Krieger, 2018).

The terrestrial species in our study showed a numerically impressive and near instantaneous attraction to the scent of conspecific death. This extreme collective attraction dwarfed the much slighter attraction shown by the marine species. Such different levels of attraction were likely driven by the fact that the terrestrial species is far more limited in its shell supply and can also roam more widely (not being constrained to tide pools), so can therefore be attracted over greater distances. Notably, the terrestrial species’ attraction to conspecific death completely outstrips any prior measures of this species’ attraction to other social stimuli, including groups of live tethered conspecifics (Laidre, 2010), jostling shells (Laidre, 2013a), or arrays of static shells (Bates & Laidre, 2018). Moreover, this species’ attraction to the smell of dead conspecifics has no element of cannibalism (Small & Thacker, 1994) and far exceeds its attraction to the smell of foods (Laidre, 2013b), thus underscoring that remodeled shells are the main, if not exclusive, sought‐after resource. Only one species of terrestrial hermit crab (the coconut crab, *Birgus latro*) does not use shells (Laidre, 2018b). This species’ predatory instincts (both on vertebrates and invertebrates) and its strong attraction to the scent of heterospecific blood (Laidre, 2017) suggest it could make an informative comparison, examining whether its level of attraction to conspecific death has been evolutionarily reduced or even lost completely since it ceased inhabiting shells.

The ultimate causes of death are myriad, though if individuals are killed two chief sources exist: predators and conspecifics. Predation on shell‐bearing organisms is a major force in the sea (Vermeij, 1978), but on land such shell predation pressure is comparatively weaker (Vermeij, 1993), and it is rarely experienced by terrestrial hermit crabs, since their remodeled shells are still outside the bite force of most terrestrial predators (Laidre, Patten, & Pruitt, 2012). Reduced predation on land might seemingly make approaching death sites safer there, but on land conspecifics effectively replace predators as arguably the most dangerous threat to one another's livelihood. Indeed, forcible eviction from one's shell by conspecifics is especially common among terrestrial hermit crabs, with coalitions of unrelated conspecifics even cooperating to evict larger individuals (Laidre, 2018c). And once an evicted individual is pulled out of the shell, its soft vulnerable abdomen (Laidre, 2007) is exposed and may be readily punctured by the claws of conspecifics as they vie for the empty shell, thereby yielding both an immediate “scent of eviction” and later a full‐blown “scent of death.” Critically, the eviction and possible ensuing killing of a conspecific generate not just one available shell, but potentially a whole series of shells, since the evictor will leave behind its prior shell after it moves into the better shell of the evictee, and so on down the line (Laidre, 2014). Such social shell exchange operates within a wider “housing market” (Laidre & Vermeij, 2012), in which individuals form so‐called vacancy chains and queue for shells by size (Bates & Laidre, 2018). Consequently, if individuals rapidly approach the scent of conspecific death, which emanates from an eviction site, they can benefit even if they are too small to move into the shell of the originally evicted individual, who may itself end up being killed. In this way, the dead may play a central role in the social systems of the living.

The transition from sea to land represents a major change in environment. However, even within the sea, subtler differences in environment may be significant in shaping responses to the scent of death. For instance, shell predation pressures within the marine environment differ dramatically across latitudes, with tropical seas experiencing much stronger forms of destructive shell predation compared to temperate seas (Bertness, Garrity, & Levings, 1981). Notably, marine hermit crabs in temperate seas are known to use death cues from shell predation events to differentiate not just the identity of the killed animal (Rittschof, 1980), but also the method of the death, being most attracted to deaths where a predator externally digests the gastropod flesh while leaving the shell intact (Valdes & Laidre, 2018). In the present study, we simulated such non‐destructive shell predation using trypsin‐treated gastropod flesh, though unlike temperate marine hermit crabs (Valdes & Laidre, 2018), the tropical marine hermit crabs in this study failed to distinguish between trypsin‐treated and pure gastropod flesh. These different response patterns between temperate and tropical marine hermit crabs may be due to the different predation pressures in temperate versus tropical marine environments. An overwhelming amount of the shell predation in the tropics involves destructive, shell‐crushing techniques (Vermeij, 1978), so tropical marine hermit crabs may therefore have few other options for locating shells than approaching both destructive and non‐destructive predation events, accepting even the damaged shells that are left after predation.

Organisms compete intensely for resources, resulting in the demise of inferior competitors. Such competition and the consequent deaths it entails are at the heart of evolution by natural selection, for the end of one individual's life indirectly clears the way for other individuals. But death can also have even more direct impacts on the living, with the end of one being's corporeal existence effectively handing off to others the very resources that fuel new opportunities. Once death and resource acquisition become so intimately connected, natural selection can favor the type of extreme collective attraction to conspecific death observed in the present study.

## CONFLICT OF INTEREST

Authors declare no competing interests.

## AUTHOR CONTRIBUTIONS

ML conceived and designed the study; LV and ML both collected the data, analyzed the data, and wrote the manuscript together.

## Data Availability

All data from this study are available as electronic supplementary material and have also been uploaded to Dryad (https://doi.org/10.5061/dryad.2t547dd).
